# Gait Recognition via Enhanced Visual–Audio Ensemble Learning with Decision Support Methods

**DOI:** 10.3390/s25123794

**Published:** 2025-06-18

**Authors:** Ruixiang Kan, Mei Wang, Tian Luo, Hongbing Qiu

**Affiliations:** 1 School of Information and Communication, Guilin University of Electronic Technology, Guilin 541004, China; bbklasnic@glut.edu.cn (R.K.); luotian@mails.guet.edu.cn (T.L.); qiuhb@guet.edu.cn (H.Q.); 2 College of Physics and Electronic Information Engineering, Guilin University of Technology, Guilin 541004, China; 3 Ministry of Education Key Laboratory of Cognitive Radio and Information Processing, Guilin University of Electronic Technology, Guilin 541004, China

**Keywords:** gait recognition, multi-sensor system, visual–audio information, ensemble learning, Gramian Angular Fields, Dempster–Shafer Evidence Theory

## Abstract

Gait is considered a valuable biometric feature, and it is essential for uncovering the latent information embedded within gait patterns. Gait recognition methods are expected to serve as significant components in numerous applications. However, existing gait recognition methods exhibit limitations in complex scenarios. To address these, we construct a dual-Kinect V2 system that focuses more on gait skeleton joint data and related acoustic signals. This setup lays a solid foundation for subsequent methods and updating strategies. The core framework consists of enhanced ensemble learning methods and Dempster–Shafer Evidence Theory (D-SET). Our recognition methods serve as the foundation, and the decision support mechanism is used to evaluate the compatibility of various modules within our system. On this basis, our main contributions are as follows: (1) an improved gait skeleton joint AdaBoost recognition method based on Circle Chaotic Mapping and Gramian Angular Field (GAF) representations; (2) a data-adaptive gait-related acoustic signal AdaBoost recognition method based on GAF and a Parallel Convolutional Neural Network (PCNN); and (3) an amalgamation of the Triangulation Topology Aggregation Optimizer (TTAO) and D-SET, providing a robust and innovative decision support mechanism. These collaborations improve the overall recognition accuracy and demonstrate their considerable application values.

## 1. Introduction

Human Activity Recognition (HAR) methods are increasingly becoming the backbone of many systems. They are widespread in domestic daily life [1,2,3,4,5,6,7,8,9,10,11,12], physical education [13,14], computer-aided medical diagnosis [15,16], human–computer interaction [17,18], and industrial production [19,20,21,22,23,24]. In this context, gait recognition on a fixed path is regarded as an unresolved issue within this domain and attracting worldwide attention.

In general, HAR systems are predominantly vision-centric, particularly in gait recognition projects. Few instances focus only on audio data. In such systems, some disadvantages cannot be ignored. On the one hand, mainstream methods primarily focus on skeleton joint data from visual inputs. Researchers worldwide are more willing to embrace deep learning approaches as the key methodology. They may typically employ Graph Convolutional Networks (GCN) [1,5,6,9,10,11], Convolutional Neural Networks (CNN) [10,14], and Attention-Based Mechanisms [8,13,21,22,23,24] as core components. While these demonstrate superior spatiotemporal feature extraction compared with traditional machine learning methods, they often exhibit many limitations in dynamic scenarios, such as gait recognition. A critical concern arises from the mandatory dimensionality reduction during their encoding or decoding phase, wherein multi-dimensional features are compressed into one-dimensional representations. This process severely constrains the modeling of spatial relationships among skeleton joints. Such inadequate approximation inevitably introduces information loss and diminishes the spatial features in particular. Although these methods might improve edge feature detection and contextual awareness, their performance often fails to cope with practical requirements in dynamic conditions. Fundamentally, the inadequate dynamic characterization and ambiguous structural relationship in joint data disrupt the systemic spatiotemporal continuity in multi-sensor systems. This leads to irrecoverable losses in fine-grained motion details [4,13]. Moreover, spatial information comprehension may conflict with processes like texture feature abstraction or contextual correlation. This issue inadvertently exacerbates cross-modal verification challenges, thereby widening the heterogeneous semantic gap. Consequently, the cross-modal mapping process in multi-sensor systems fails to adequately accommodate multi-scale feature perceptions [1,8,13,17,18]. So, it potentially leads to increased misclassification rates. These drawbacks significantly hinder the vision-dominant gait recognition methods in complex scenarios. This means context-aware integration of visual–audio information analysis is needed [5]. Among them, the Transformer-based approach is an essential component of attention-based methods. Undoubtedly, these approaches have become the mainstream techniques for high-dimensional skeleton joint recognition. They provide a more suitable research framework for subtle clue comprehension. In terms of multi-scale gait features, these methods excel at capturing long-range relationships, thereby highlighting the implicit associations among core skeleton joints. However, it is important to acknowledge that these methods also have their limitations. Specifically, the improper compression of spatial information is a weak point within Transformer-based approaches. This shortcoming can negatively impact the retention of core spatial information in low-dimensional feature representations, which is detrimental to the analysis of skeleton joint sets. Additionally, the significant computational complexity associated with these methods further limits their applicability. Notably, the self-occlusion issues suffered from Kinect families (Microsoft Corp., Redmond, WA, USA), Leap Motion Controller families (LMC, Leap Motion Inc., San Francisco, CA, USA), and some other tools exemplify these challenges in real-world applications [3,4,17]. These unresolved issues demand urgent attention, and more flexible solutions for recognition models are necessary.

On the other hand, the drawbacks arising from insufficient dynamic characteristic understanding in gait recognition are becoming increasingly evident in audio-centric research. Although acoustic signals may not always be regarded as the core of multi-sensor systems, they undoubtedly play a critical role in gait recognition. Historically, these studies have been affected by several weaknesses [3,4,15,17,18]. Specifically, conventional acoustic recognition approaches lack comprehensive contextual information or periodic variation pattern analysis, which isolates them from visual data. Furthermore, they fail to incorporate adaptive evaluation mechanisms for scenario compatibility. In response to the above phenomenon, this study implements a vision-centric, audio-assisted framework that necessitates an appropriate decision support mechanism. In this context, the D-SET turns out to be a viable solution.

However, existing decision support mechanisms in multi-sensor systems also exhibit lots of limitations. They fail to account for the diversity of evidence descriptions and granularity differences in our system, resulting in a passive approach while neglecting to facilitate appropriate cross-modal representation. Particularly in scenarios influenced by huge disparities in evidence support degree contributions, they may struggle to address the “pattern collapse” issue encountered under extreme conditions [19]. Furthermore, the heterogeneous nature of the evidence presents considerable challenges for multi-sensor decision support, while current systems often lack the necessary adaptive adjustment mechanisms in these situations. This long-standing deficiency is gradually becoming evident in visual–audio recognition research. To tackle these issues, our study implements an enhanced framework that combines the AdaBoost model with an improved D-SET-based decision support mechanism. On this basis, our main contributions are as follows:

(1) To cope with insufficient spatial feature comprehension, weak periodic feature extraction, and conflicting multi-scale gait information perception, we implement several enhancements in our visual information recognition module. Firstly, we propose a Circle-Chaotic-Mapping-based method to optimize base classifier initialization, thereby enhancing nonlinear gait feature understanding. Secondly, we develop a GAF-based multi-input graph representation method that lies at the core of the various base classifiers within the AdaBoost model. This approach effectively interfaces with the subsequent Bidirectional Gated Recurrent Unit (BiGRU) and Bidirectional Long Short-Term Memory (BiLSTM) structures, while also improving the multi-scale dynamic characteristic understanding and the asymmetry perception across different gait periods. Finally, experimental results demonstrate a recognition accuracy of 99.01% across four key gait visual information recognition tasks.
(2) Within the multi-sensor system framework, the audio information recognition module serves as a complementary solution to the visual information recognition module in adverse conditions. As for inadequate comprehension of non-stationary characteristics, insufficient response to periodic variations, and poor generalizability of semantic features, we propose an enhanced gait-related acoustic signal recognition method. This method combines Hilbert-Transform-based periodicity enhancement with improved GAF-based methods and PCNN architecture. Subsequently, a dual-input structure is adopted for the form base classifiers in the AdaBoost model. Experimental results demonstrate a recognition accuracy of 97.75% across four gait-related acoustic signal recognition tasks.
(3) To address the shortcomings in the applicability analysis of multi-sensor modules and to mitigate the “pattern collapse” in D-SET, we propose a novel strategy that integrates an improved TTAO into the evidence recombination process [25]. This approach enhances the adaptive weighting of related recognition modules in our system. Our decision support mechanism incorporates a quantitative analysis of both mass function optimization and evidence recombination processes. It provides a reliable global module adaptability analysis in challenging environments. Experimental results on eight challenging gait visual–audio recognition tasks demonstrate a considerable improvement. It achieves a recognition accuracy of 98.44% and surpasses current state-of-the-art solutions.

The rest of this manuscript is organized as follows. Section 2 presents the fundamental theories underlying the visual–audio recognition methods and decision support mechanism within our system. Section 3 describes the system setup and some necessary processing steps. Building on these foundations, Section 4 provides a detailed discussion of the updating strategies for the visual information recognition module, the audio information recognition module, and the decision support mechanism. Section 5 reveals comprehensive experiments using the gait recognition methods, emphasizing the synergistic collaboration between the decision support mechanism and recognition methods. They are followed by an objective analysis. Section 6 summarizes our work. Finally, the manuscript concludes with potential future work and directions.

## 2. Related Work

### 2.1. AdaBoost Methods

Skeleton joint recognition in HAR systems has become increasingly rigorous. In single-person scenarios, the focus has shifted from conventional features, such as contour information and texture characteristics, to a greater emphasis on abstract attributes and high-frequency variations. At the same time, researchers worldwide have progressively concentrated on aspects that better reflect implicit patterns. This is a warm tide particularly evident in gait recognition projects [5,6,7,8,12,21]. In addition, feature-level and set-level processing in multi-sensor systems now play pivotal roles. However, current deep learning methods, such as CNN-based, Recurrent Neural Network (RNN)-based, GCN-based, and attention-based methods, are having a hard time handling multi-scale and periodic gait features. Specifically, CNN-based models truly excel at extracting local features based on hierarchical convolution operations. However, their layer-wise processing inadvertently diminishes the spatial correlation characteristics essential for gait analysis [2,14,21]. RNN-based models effectively process temporal gait sequences but often struggle to capture multi-periodic spatial patterns [3,17]. GCN-based models take a bird’s eye view of the topological structures of joints while bringing reduced sensitivity to subtle low-level gait feature variations [6,9,11]. Lastly, attention-based models lack flexibility in periodic gait analysis. They also struggle to simultaneously ensure multi-scale feature perception or spatial structure comprehension [7,8,9,10,11,13,21]. They are supposed to integrate with other methods to become a more reasonable solution. It is essential to balance low-level feature and high-level feature perception in gait analysis effectively [5,6,16]. Inspired by these findings, the ensemble learning method serves as a proper solution.

Ensemble learning methods enhance predictive performance beyond that of individual base classifiers by strategically combining multiple weak classifiers into a strong classifier. The taxonomy of ensemble learning methods categorizes them into three main paradigms based on their construction logic: Boosting, Bagging, and Stacking. The Boosting logic employs an iterative training process with adaptive weight adjustment, where plenty of base classifiers are adjusted to correct the errors made by previous models, thereby progressively improving overall performance [4,26,27]. In contrast, the Bagging logic implements bootstrapping processes from the entire training set when necessary, with final predictions determined via voting mechanisms across independently trained classifiers [28,29,30,31,32]. The Stacking logic adopts a hierarchical architecture that integrates heterogeneous base classifiers through multi-stage input processing. This approach utilizes a meta-learner to synthesize the outputs from primary classifiers and subsequently generates dynamic predictions based on required combination rules [29].

Therefore, the AdaBoost method emerges as the optimal choice for gait recognition. On the basis of suitable base classifiers, it can achieve efficient multi-scale feature perceptions and steadily analyze subtle clues. In the general case of this method, N is used as the total number of training samples, and $y_{i}$ represents the actual output in the current iteration. During the t-th iteration, the classification error is calculated based on the weight $w_{t}$ to be updated and the base classifier $h_{t}$. Judging by the start and end conditions, the loop continues when necessary. I(⋅) is denoted as the indicator function. Its value equals 1 when the condition is true and 0 otherwise. During the t-th iteration, the classification error $\varepsilon_{t}$ and the weight distribution $w_{t+1}$ to be calculated can be expressed as

(1) $$\left\{\begin{array}{c} \varepsilon_{t}=\sum_{i=1}^{N}w_{t}(i)\cdot I[h_{t}(x_{i})\neq y_{i}]\\ \alpha_{t}=\frac{1}{2}\ln(\frac{1-\varepsilon_{t}}{\varepsilon_{t}})\\ w_{t+1}(i)=w_{t}(i)\cdot \exp[-\alpha_{t}\cdot y_{i}\cdot h_{t}(x_{i})]\\ \sum_{i=0}^{N}w_{t+1}(i)=1 \end{array}\right.$$

The weights are dynamically updated in each iteration and normalized. The final model H(x) is formed through a weighted combination mechanism. T is set as the maximum number of iterations, sign(⋅) represents the sign function, and $\alpha_{t}$ indicates the weight of one base classifier. This process can be described as

(2) $$H(x)=sign[\sum_{t=1}^{T}\alpha_{t}\cdot h_{t}(x)]$$

Consequently, we incorporate gait characteristics [5,6,15,16] to develop both visual and audio recognition modules. The model designed specifically addresses three critical aspects: (1) parameter initialization in AdaBoost, (2) error iteration methodology, and (3) multi-scale comprehension of visual–audio information. It establishes a stable foundation for robust gait visual–audio information recognition in real-world situations.

### 2.2. D-S Evidence Theory

As for gait recognition on a fixed path, there is still a huge gap. It often exists in the availability of comprehensive decision support frameworks. They are expected to be capable of quantitatively evaluating the effectiveness of various recognition modules. In this regard, conducting in-depth research grounded in the D-SET represents a well-justified solution [33,34,35,36].

The D-SET is widely applied in multi-sensor systems. It often begins with the construction of evidence bodies, and then it quantifies the credibility of individual evidence through Basic Probability Assignment (BPA) calculations and conflict factor analysis to facilitate decision making. The support degree of multiple evidence sources is determined according to context-specific Evidence Combination Rules during this moment, which leads to the mass function and the conflict factors calculation. The final step involves adjusting the BPA allocation based on the conflict factors, and it brings optimized adaptive weights for each module in our system. During this period, evidence recombination and gradient descent optimizations collectively enhance both local optimization and global search capabilities. In our system, the visual information recognition module serves as the default recognition method. K is used as the conflict factor. A represents the set of module applicability degrees processed by the decision support mechanism. B represents the initial support degree allocation of the visual information recognition module, and C represents the initial support degree allocation of the audio information recognition module for the same task. We define $m_{v}$ as the BPA of the visual recognition module and $m_{a}$ as the BPA of the audio recognition module. Θ encompasses all possible module applicability combinations. m(Θ) quantifies the support degree of the evidence body set, which measures system uncertainty tailored to gait recognition. Bel(⋅) is used as the belief function. These processes can be described as

(3) $$m(\Theta )=\frac{\sum_{B\subseteq A,C\subseteq A}m_{v}(B)m_{a}(C)}{1-K},Bel(A)=\sum_{B\subseteq A}m(B),K=\sum_{B\cap C\neq \emptyset }m_{v}(B)m_{a}(C)$$

This framework quantitatively assesses the conflict degree within the system. We further propose a novel criterion for evaluating conflict coefficient K in D-SET. When K≥0.3, the scenario is identified as exhibiting a significant disparity in evidence support, thereby necessitating intervention from the decision support mechanism to mitigate potential “pattern collapse” issues. Conversely, in scenarios without a huge disparity in evidence support, our system proceeds according to the default configuration.

The adaptive weight adjustment and recombination for multiple evidence bodies can dynamically enhance the capability of multi-sensor information processing in our system. With the help of recognition modules, this framework facilitates the adaptive processing of multi-source heterogeneous information within the multi-sensor system, thereby indirectly improving model robustness. By bridging the huge gap between the visual and audio information recognition modules in our system, the decision support mechanism can quantitatively mitigate or cope with the negative impacts in adverse conditions. The evidence body $E_{v}$ from the visual recognition module and evidence body $E_{a}$ from the audio recognition module are combined with their initial weights, $w_{v}$ and $w_{a}$, respectively. The learning rate η=0.01. Assuming θ represents scenario-adaptive prior conditions, the updating process for $E_{v}$ and $E_{a}$ via a gradient descent formula during the u-th iteration can be described as

(4) $$\begin{array}{l} \nabla f(w_{v}^{\left(u\right)})=\frac{\partial \log P(E_{v}|\theta )}{\partial w_{v}^{\left(u\right)}},\nabla f(w_{a}^{\left(u\right)})=\frac{\partial \log P(E_{a}|\theta )}{\partial w_{a}^{\left(u\right)}}\\ w_{v}^{\left(u+1\right)}=w_{v}^{\left(u\right)}-\eta \nabla f(w_{v}^{\left(u\right)}),w_{a}^{\left(u+1\right)}=w_{a}^{\left(u\right)}-\eta \nabla f(w_{a}^{\left(u\right)}) \end{array}$$

In summary, we integrate the D-SET with the AdaBoost model to establish a framework that synthesizes outputs from diverse classifiers and enhances classification performance through quantitatively driven decision support. Our framework addresses not only periodic and multi-scale feature learning but optimizes the adaptive weight adjustments, as well. On this basis, the three core modules operate synergistically to achieve more robust gait recognition in the real-world situation.

## 3. System Setup

### 3.1. Inter-Module Collaboration Analysis

**Figure 1:**
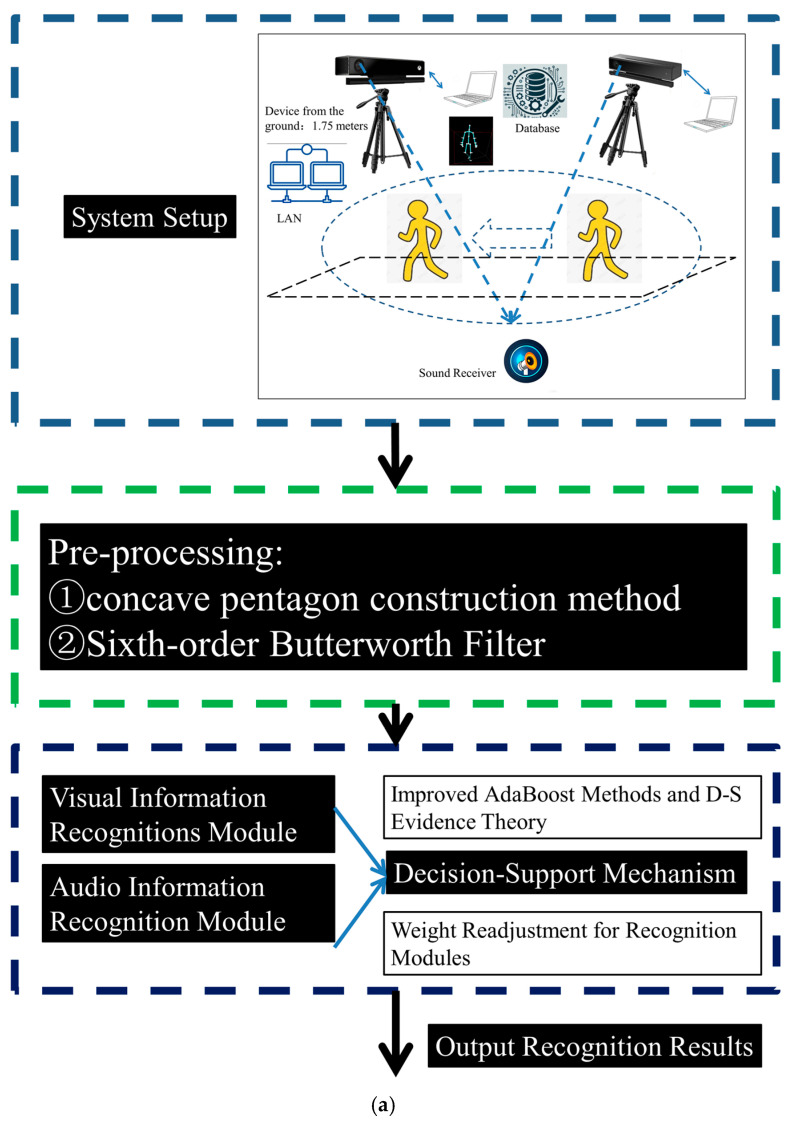

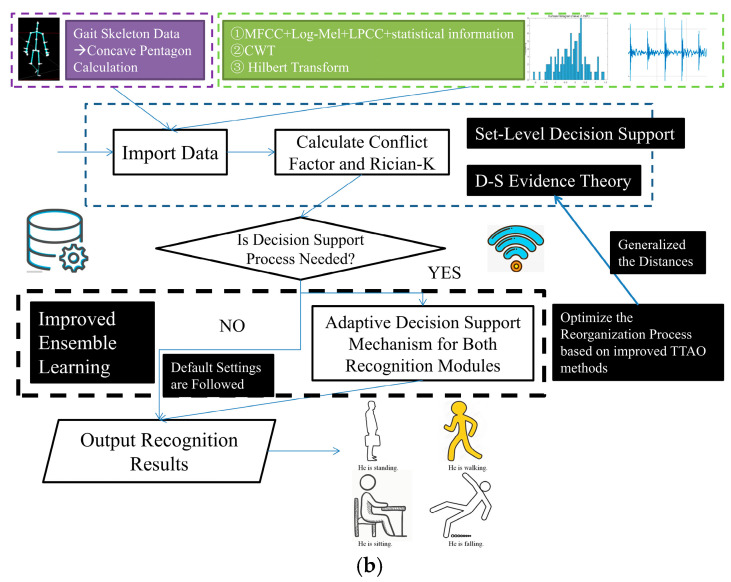
System framework and working flow. (**a**) System framework. It is used to describe the relationship among different modules. (**b**) System working flow. It is used to describe the decision support logic.

Our system is constructed by using dual-Kinect V2 sensors, and it incorporates the multi-sensor data transmission protocol from [4] to settle TCP packet concatenation issues. In our system, the Kinect V2 is adopted to capture skeleton joint data. Subsequent data preprocessing occurs on the server, where visual and audio information recognition is conducted when necessary. Notably, the Kinect V2 is of great value in our system. Its integrated infrared module significantly enhances our ability to perform gait recognition that is minimally affected by lighting conditions. Furthermore, the effective range of the Kinect V2 is adequate to completely encompass the walking area of the participants. However, it is evident that the skeleton joint data obtained from the Kinect V2 system has its limitations, particularly concerning self-occlusion issues in scenarios with flexible orientations. Undoubtedly, these irregular and random connections are detrimental to human action recognition and gait recognition. To mitigate the self-occlusion problem, we employ a circular arrangement scheme in our dual-Kinect V2 system, which has been previously validated as advantageous for recognition tasks based on skeleton joint data. When skeleton joint data is heavily occluded, our system may require further adjustments to the height of the Kinect V2 from the ground, as well as to the pitch angles of the device. These measures significantly reduce the occlusions in such scenarios. At the same time, it is important to emphasize that our system is greatly influenced by the performance characteristics of the Kinect V2 device, including factors like noise resistance and the reception range of skeleton joints. Before the official Kinect V2 undergoes a large-scale upgrade, our system can be used within its operational range effectively. In our system, the users are supposed to engage within a range of 4 m from the Kinect V2 device. Based on actual measurements, it can accommodate usage within a 4 m × 2 m area or a 3 m × 3 m area. When considering power consumption for connecting two Kinect V2 devices, the total power for this system does not exceed 800 W. In terms of power consumption, this system does not have any special requirements. According to the standards in China Mainland, a voltage of 220 V is necessary. If used in Taiwan Province, an additional voltage converter is required to adapt to the 110 V environment. The visual information recognition module is given higher priority, while the audio recognition module serves as a fallback solution for vision-compromised scenarios. The decision support mechanism further enhances module adaptability through quantitative analysis that emphasizes prior probabilities. Referring to [4,5,9,15,16,37,38], our system’s framework and its working flow are presented below (Figure 1).

On this basis, we can provide a more detailed description of the connections and relationships among the various modules in our system.

As for the visual information recognition module, we have optimized the initialization process of the AdaBoost model using Circle Chaotic Mapping [39]. It is adopted to enhance its ability to capture nonlinear features during subsequent training. Furthermore, a GAF-based multi-input representation approach has been employed across the various modules, which enhances the distinguishability of gait skeleton joint data while improving the time–frequency–spatial interconnection analysis among different gait periods under specific conditions. At the same time, it is significant to note that the feature extraction methods employed not only emphasize variations in spatial structures but preserve the dynamic characteristics influenced by gait phases, as well. These measures lay a solid foundation for the effectiveness of the subsequent AdaBoost model and the multi-input graphical representation-based classifier structure.

As for the gait-related acoustic signal recognition module, the PCNN and its dual-input structure have also been adapted to the GAF-based multi-input approach. It becomes the foundation of the base classifier within this module. At the same time, feature extraction processes along with the Hilbert Transform are employed to highlight envelope information. They can further underscore the benefits of the GAF-based graphical representation approach, which emphasizes the patterns of energy spectrum variations. Additionally, the statistical characteristics of specific nodes across various gait periods are further accentuated, thereby enhancing the performance of the classifier.

Recognizing the inherent limitations of each recognition module, we have introduced a decision support mechanism based on D-S Evidence Theory. This mechanism is designed to enable the system to select the most suitable recognition module for gait recognition in complex scenarios. It is intended to score the adaptability of each core module in this scenario based on the weight distribution readjustments from the decision support mechanism. On this basis, it can help our system complete the related recognition process accordingly.

Last but not least, due to the limitations of D-S Evidence Theory in scenarios characterized by significant discrepancies and heterogeneity among various evidence, we have generalized the concept of distance between different forms of evidence and introduced TTAO to enhance the readjustment process accordingly. We believe that the generalized distance statistics of the evidence bodies objectively reflect the adaptability comparisons of each module within the current context. This makes our decision support mechanism quantitatively assess the adaptability of each module within our system and adjust the overall system processes accordingly when necessary. This improvement enables the decision support mechanism to be more targeted, allowing the system to effectively utilize the recognition module in advantageous conditions for gait recognition under these circumstances.

### 3.2. Feature Extraction and Preprocessing Methods

**Figure 2:**
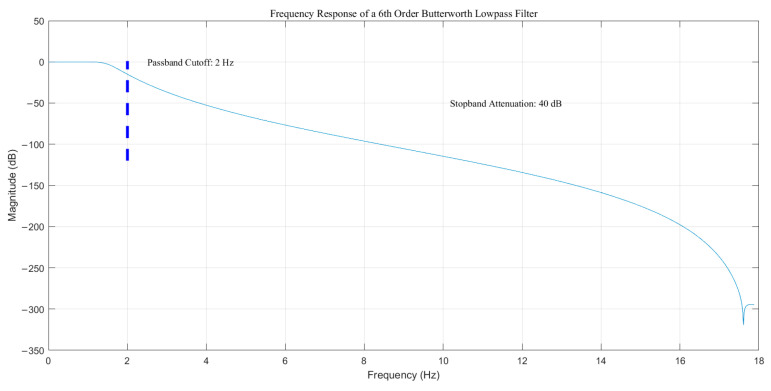
Sixth-order Butterworth filter.

We adopt the methodology from [4,36] to eliminate noises during our experiment. Subsequently, a sixth-order Butterworth filter is configured with the following settings: passband cutoff frequency at 2 Hz, maximum passband attenuation at 1dB, and stopband attenuation at 40 dB. It can be shown as follows (Figure 2).

The system employs Euclidean distances between the bilateral elbow and knee joints to identify key frames within gait periods, enabling precise determination of initiation and termination frames while highlighting the differences between Stance and Swing phases [16]. This approach concurrently addresses two pivotal aspects of gait analysis: spatial information comprehension and periodic variation characterization. Referring to [4], we integrate (1) lower-body kinematic features, (2) distance metrics derived from Euclidean distances and vector projections, (3) angular characteristics, and (4) nine dynamic triangular areas. In addition, the framework incorporates a concave pentagon construction method that analyzes the dynamic relationships between the spine’s base and spine shoulder joints. On this basis, we calculate the perimeters and areas of the two related concave pentagons. They can enhance the correlation between spatial information and dynamic characteristics. For single-subject scenarios, the distance dis between any two coordinates $P_{1}(x_{1},y_{1},z_{1})$ and $P_{2}(x_{2},y_{2},z_{2})$ can be calculated as follows:

(5) $$dis=\sqrt{{\left(x_{2}-x_{1}\right)}^{2}+{\left(y_{2}-y_{1}\right)}^{2}+{\left(z_{2}-z_{1}\right)}^{2}}$$

For an arbitrary concave pentagon, it may contain $P_{1}$, $P_{2}$, $P_{3}$, $P_{4}$, and $P_{5}$. If $P_{3}$ denotes the reflex vertex, the perimeter L can be calculated as follows:

(6) $$L=dis(P_{1},P_{2})+dis(P_{2},P_{3})+dis(P_{3},P_{4})+dis(P_{4},P_{5})+dis(P_{5},P_{1})$$

By sequentially incorporating the position of the reflex vertex, the concave pentagon can be partitioned into three triangles. $T_{1}$ consists of $P_{1},P_{2},P_{3}$. $T_{2}$ consists of $P_{2},P_{3},P_{4}$. $T_{3}$ consists of $P_{3},P_{4},P_{5}$. It helps to illustrate the geometric properties by using vector cross products. The area of it is then adopted for the feature and can be expressed as

(7) $$\begin{array}{l} Area_{Polygon}=Area_{T_{1}}+Area_{T_{2}}+Area_{T_{3}}\\ =\frac{1}{2}(\left\|\left(P_{2}-P_{1})\times (P_{3}-P_{1}\right)\right\|+\left\|\left(P_{3}-P_{1})\times (P_{4}-P_{1}\right)\right\|+\left\|\left(P_{4}-P_{1})\times (P_{5}-P_{1}\right)\right\|) \end{array}$$

In addition, the limitations of the Kinect V2 in receiving audio information result in inevitable multitrack compression, which leads to the loss of spatial information in the captured acoustic signals. Therefore, we do not employ the non-uniform linear microphone array of the Kinect V2 as the audio receiving device. Although the audio information recognition module in our system is not assigned a high priority, its enormous role in vision-compromised scenarios remains undeniable. As for acoustic signals within the system, we have enhanced the feature extraction methods from [23,24]. Mel-Frequency Cepstral Coefficients (MFCC), Log-Mel spectrograms, and Linear Predictive Cepstral Coefficients (LPCC) are needed to construct feature sets and conduct frame-by-frame time-frequency analysis. However, existing approaches have a difficult time analyzing the periodic variation trends of multi-sensor information. Some participants under load bearing or suffering from other illnesses often exhibit limited capacity of muscle control under this circumstance. This situation makes it challenging to maintain periodic gait normalization. To address this issue, we incorporate several useful statistical features. They are kurtosis, skewness, and the Rician-K factor. They are used to enhance the perception of statistical characteristics, which proves particularly advantageous for abnormal gait recognition. Firstly, gait-related acoustic signals under this circumstance may exhibit sharp components unexpectedly. The kurtosis feature exactly captures these variations in acoustic signals relevant to gait recognition. Secondly, some factors mentioned above frequently lead to asymmetry in abnormal gait patterns. Thus, incorporating skewness to quantify this asymmetry is essential. This frame-by-frame analysis combined with these statistical indicators objectively amplifies the distinguishability of abnormal gait acoustic signals. n is used as the total number of samples and $x_{i}$ represents the i-th discrete feature point. $\bar{x}$ represents the sample mean value. s represents the sample standard deviation value. A correction term is adopted to mitigate bias in sample estimations under this circumstance. For one gait acoustic signal sequence after sampling, the kurtosis Ku and the skewness Sk can be calculated as follows:

(8) $$\begin{array}{l} Ku=\frac{n(n+1)}{\left(n-1)(n-2)(n-3\right)}\sum_{i=1}^{n}{\left(\frac{x_{i}-\bar{x}}{s}\right)}^{4}-\frac{3{\left(n-1\right)}^{2}}{\left(n-2)(n-3\right)}\\ Sk=\frac{n}{\left(n-1)(n-2\right)}{\sum_{i=1}^{n}\left(\frac{x_{i}-\bar{x}}{s}\right)}^{3} \end{array}$$

We further adopt the Rician-K factor to refine the process, enabling precise characterization of the relationship between gait-related acoustic signals and noise intensity. υ is used as the amplitude of the target signal component and σ represents the standard deviation of the noise component. This factor can be described as follows:

(9) $$R\_K=\frac{\upsilon^{2}}{2\sigma^{2}}$$

In real-world scenarios, a high degree of kurtosis combined with a low R_K value indicates that significant interference occurs within the system. Meanwhile, a high degree of skewness combined with a high R_K value indicates that significant asymmetry is appearing during gait periods. They can be used to illustrate abnormal gait patterns or associated events. Additionally, the Hilbert-Transform-based envelope analysis of gait signals is also applied to enhance the detection of period boundaries in dynamic environments. With these preprocessing stages for visual–audio information established, our system employs its decision support mechanism to adjust multi-module processing for robust human gait recognition in complex scenarios (noise level of less than 50 dB).

## 4. Updating Strategies for Different Modules in Our System

### 4.1. Gait Skeleton Joint AdaBoost Recognition Based on Enhanced Chaotic Mapping and GAF Methods

Gait skeleton joint recognition methods are encountering diminishing effectiveness in complex scenarios due to three critical limitations: improper learning patterns, ambiguous training iteration tendencies, and insufficient targeted weight adjustment. These constraints do more harm than good within our system. Therefore, effective measures are needed in our visual information recognition methods. We focus on enhancing the AdaBoost model to simultaneously capture three key aspects of skeleton joint data: spatial structural characteristics, flexible subtle features, and periodic intensity variations in time. For gait recognition, the effective multi-scale representation of skeleton joints is a prerequisite for the AdaBoost model to achieve more accurate recognition results. There is no doubt that the GAF-based multi-input graph representation approach meets our requirements [40]. On the one hand, it provides a comprehensive understanding of skeleton joint data by capturing subtle clues during frame-by-frame analysis. On the other hand, the analysis of the energy spectrum highlights the asymmetrical characteristics present in various gait phases. They serve as a crucial reference for the modules within our system. Both aspects are essential for our algorithm, which is why we have adopted a dual-input structure for system construction.

To provide a more precise description of the dual-input base classifiers, more details should be given. The upper branch (GASF-BiGRU branch) generates a 120 × 120 single-channel expression by using GASF, which is then flattened row-wise into a sequence. Subsequently, temporal feature extraction is carried out using a two-layer BiGRU network with 64 hidden units. The GASF representations are adopted here for asymmetry modeling and periodic characteristic variations, thereby enabling a more reasonable focus on dynamic patterns. Following this, global average pooling is applied to the output sequence of the BiGRU along the temporal dimension. This operation reveals the overall trend of the time series while reducing computational complexity to some extent. In the lower branch (GEF-BiLSTM branch), the same serialization strategy is applied to process the GEF representation, which is then fed into a two-layer BiLSTM network (also with 64 hidden units). This also leverages the gating mechanism to accommodate the long-range dependencies inherent in the statistical characteristics of the energy field under this circumstance. Next, global max pooling is performed on the sequence to extract a 128-dimensional feature set, highlighting the local extrema of energy distribution.

Subsequently, the model concatenates the 128-dimensional features from two branches and employs a four-head attention mechanism (with a key/query dimension of 64 and independent parameters between heads) to adaptively integrate cross-branch relational analysis. Finally, a fully connected layer maps the 256-dimensional features to an 8-dimensional output for class probability distribution using the Softmax function. In total, the trainable parameter count for the base classifier is approximately 0.6 M, with FLOPs amounting to 0.06 billion. At the same time, an early stopping strategy is employed for the AdaBoost implementation, with the maximum number of iterations set to 15. Consequently, the total trainable parameter count for the model is approximately 2.7 million, with FLOPs amounting to 0.3 billion.

**Figure 3:**
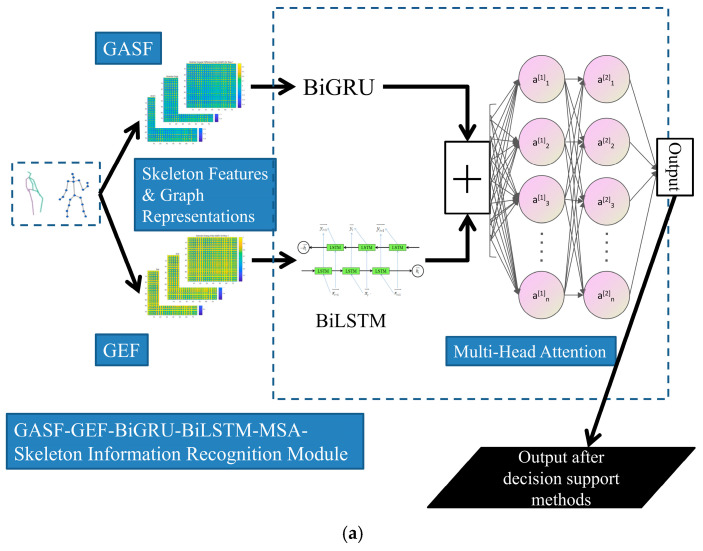

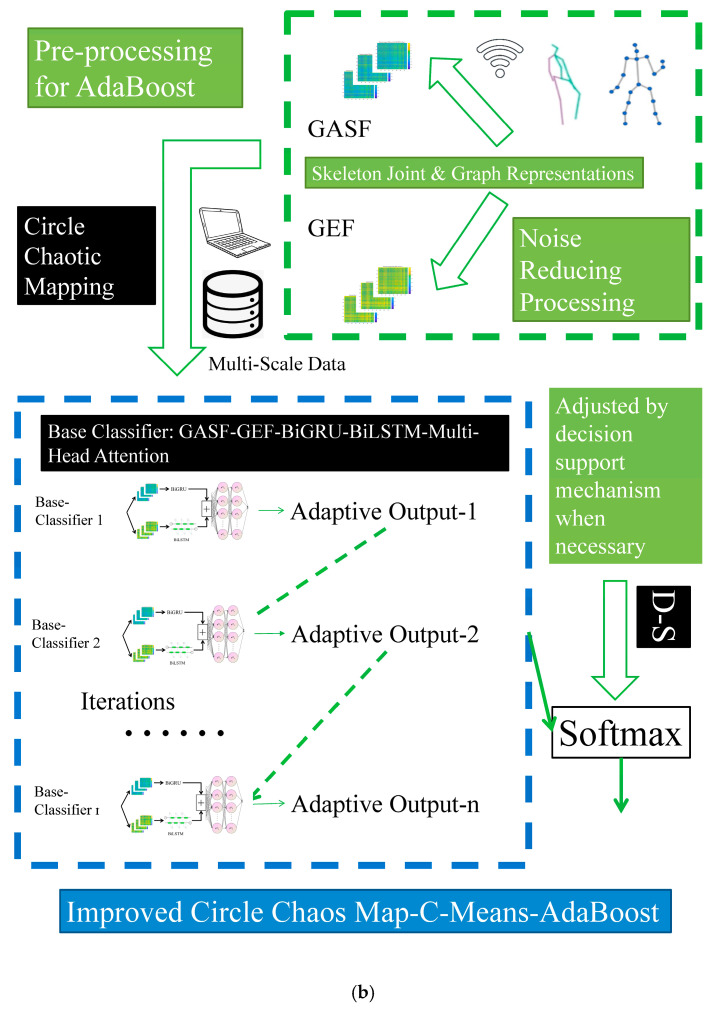
Visual information recognition module in our system. (**a**) Base classifier framework for visual information recognition module. (**b**) Improved AdaBoost method for visual information recognition module.

In terms of the visual information recognition module in our system, the base classifier framework and the entire improved AdaBoost recognition model are presented in Figure 3a and Figure 3b, respectively.

On the one hand, a novel strategy utilizing Circle Chaotic Mapping is adopted to optimize base classifier initialization. Human gait exhibits a periodic nature. In this context, we employ Circle Chaotic Mapping to generate a more diverse initial weight distribution. This enhancement enables the model to explore the parameter space more effectively during the training process and facilitates a timely global search in specific situations. For skeleton joint data, diversity is introduced into the feature spaces through perturbations or reorganizations derived from the internal mechanisms of Circle Chaotic Mapping. This approach not only captures the general patterns among the skeleton joints but also reveals clues that are inherently concealed within the gait multi-scale features. Consequently, it ensures that the AdaBoost model achieves improved exploratory capabilities. These factors may also exert a significant influence on the initialization process. By utilizing Circle Chaotic Mapping, we establish more reasonable initial weight distribution during the weight initialization phase. This enhancement enables AdaBoost models to better adapt to the complexity of the training data throughout the training process, thereby reducing the risk of overfitting to some extent. Ultimately, this allows AdaBoost models to converge more quickly and accurately to the optimal classification boundary during training. This approach widely strengthens nonlinear feature understanding in gait skeleton joint analysis. The improved nonlinear mapping relationships not only strengthen the effectiveness of initialization in combination with parameter optimization processes but also reduce the risk of converging to local minima, as well. On this basis, our method showcases better adaptability to error-based judgment processes during subsequent iterations.

On the other hand, to deal with the inefficient understanding between set-level and frame-level features in gait skeleton joint data, we adopt a novel multi-input feature extraction method that integrates one-dimensional discrete time-series joint tracking data and dual-input enhanced GAF-based graph signal representations from each gait period [40]. We believe that during their abnormal gait periods, the subjects’ inherent limitations in muscle control may result in passive, irregular tremors and vibrations during their gait patterns. Focusing more on skeleton joint data, these asymmetrical characteristics will be reflected in the energy spectrum and frame-by-frame correlation coefficients. Consequently, we introduce a dual-input structure guided by a GAF-based approach. They are expected to be used in the base classifiers to enhance abnormal gait multi-scale feature perception. For GAF processing, gait visual–audio information first undergoes normalization. Related angular relationships between the dimensions of the discrete sequence are then calculated to establish inter-frame similarity measures. It is bound to generate bijective mapping through polar coordinate transformation and vector inner product computation. $f_{s}$ represents a specific feature in the sample set, H denotes the number of features to be processed, and G represents the Gram matrix. It can be shown as

(10) $$\begin{array}{l} {\tilde{f}}_{s}=\frac{f_{s}-\min(f)}{\max(f)-\min(f)}\times 2-1\\ \phi_{s}=\arccos({\tilde{f}}_{s})\\ G=\left[\begin{array}{cccc} \cos(\phi_{1,1}) & \cos(\phi_{1,2}) & \ldots & \cos(\phi_{1,H})\\ \cos(\phi_{2,1}) & \cos(\phi_{2,2}) & \ldots & \cos(\phi_{2,H})\\ \vdots & \vdots & \ddots & \vdots \\ \cos(\phi_{H,1}) & \cos(\phi_{H,1}) & \ldots & \cos(\phi_{H,H}) \end{array}\right] \end{array}$$

They are presented to amplify graph representations [40]. Extending these approaches, corresponding matrices can be obtained through angular difference and angular summation operations, respectively. Then, two methods are developed based on the inter-frame difference approach in the polar coordinate system. They are the Gramian Angular Difference Field (GADF) and the Gramian Angular Summation Field (GASF). The GADF method provides a refined similarity measurement between adjacent frames. It energizes the essential descriptions of key frames through two-dimensional similarity representations. Meanwhile, the GASF method reveals global temporal relationships within the time series. It proves to be effective for capturing flexible gait information [4,40,41]. Both representations are optimally utilized according to gait visual–audio data characteristics. For the i-th and j-th processing stages within a gait period, the GADF and GASF methods can be expressed as

(11) $$\left\{\begin{array}{c} \Delta \phi_{s}=\phi_{s+1}-\phi_{s}\\ GADF[i,j]=\sin(\Delta \phi_{i}+\Delta \phi_{j})\\ GASF[i,j]=\cos(\phi_{i}+\phi_{j}) \end{array}\right.$$

This framework makes the base classifiers simultaneously grasp low-level features (e.g., spatial information and contour characteristics) while emphasizing contextual awareness and semantic feature abstraction in complex scenarios. It also establishes a solid foundation for leveraging the dual-input structural advantages of base classifiers across different modules [7]. We further introduce a novel Gramian Energy Field (GEF) graph representation to illustrate the differentiation of abnormal gait patterns. The GEF method proves effective for skeleton joint recognition or analysis. In connection with Equations (10) and (11), it can be described as

(12) $$GEF[i,j]=GADF^{2}[i,j]+GASF^{2}[i,j]$$

**Figure 4:**
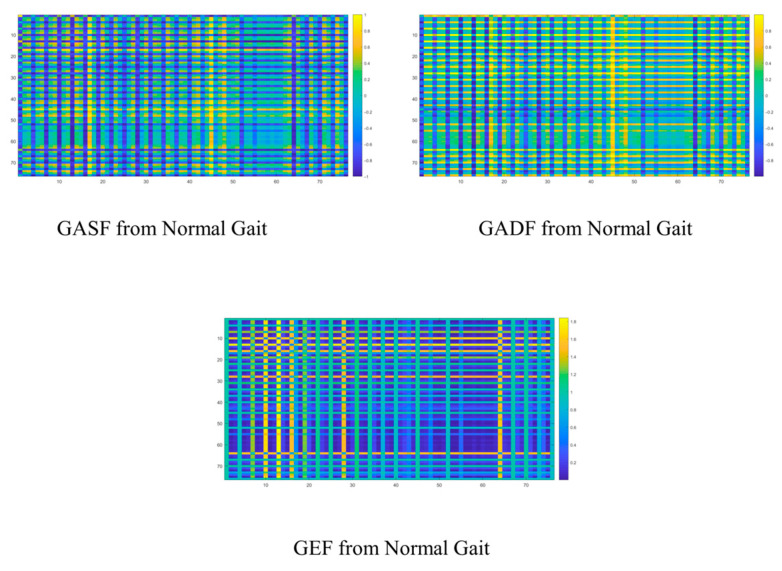
Normal gait GAF-based graphs.

There is no doubt that the graph representations here further elucidate relevant abstract clues. Firstly, the frame-by-frame analysis process inherent in the GAF-based graph representation methods allows us to capture additional temporal–frequency spatial coupling characteristics associated with abnormal gait. These features will assist AdaBoost in achieving more comprehensive multi-scale feature comprehension. Secondly, the multi-input structure of the base classifiers objectively enables the model to effectively integrate the entire process. Thirdly, the energy spectrum distribution and periodic GEF analysis derived from this study represent crucial elements that cannot be overlooked. Objectively, the statistical characteristics of the energy spectrum obtained through this frame-by-frame analysis provide a more precise quantification of the asymmetry that appears during various gait periods. In the spectral representation, the frequency differences and statistical relationships of high-energy nodes will offer valuable insights to the recognition modules within our system. More simulations are carried out by using the GIST dataset from [5]. Applying the proposed GAF-based method, the corresponding GAF-based graphs for the normal gait are shown below (Figure 4).

**Figure 5:**
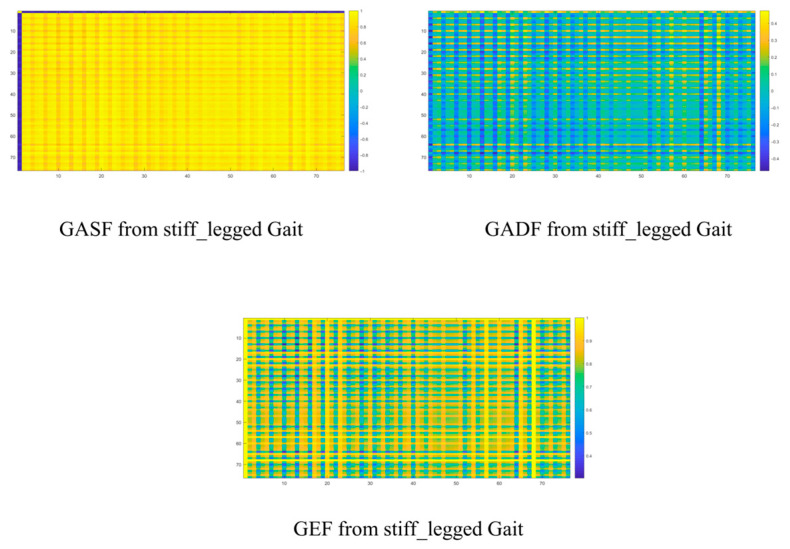
Stiff-legged gait GAF-based graphs.

Regarding the stiff-legged gait, the GAF-based approach quantitatively and visually reveals the limitations of conventional methods in terms of periodic feature perception or spatial structure awareness. These deficiencies are evident in the joint representation patterns across various rows and columns, where the system highlights both the asymmetries characteristic of abnormal gait and the difference within skeleton joint distributions in time. There is no doubt that lots of abnormalities exist in the movement patterns of core skeleton joints due to patients’ diminishing muscle control abilities. The feedback addressing these abnormalities in the graph representation approach is reflected in the differences in energy spectrum distribution, as well as the variations resulting from frame-by-frame analysis. These factors are expected to positively influence the recognition modules within the system. They can be shown as follows (Figure 5).

It is apparent that emphasizing statistical features further enhances the differentiation among different types of gaits, particularly expanding energy distribution differences. In summary, this visual information recognition module successfully employs an AdaBoost-based framework augmented by Circle Chaotic Mapping during the initialization process. Dual-input base classifiers are implemented to achieve robust multi-scale features in conjunction with GAF-based processing. The final output stage utilizes a multi-head attention mechanism for adaptive feature readjustment. It helps our method obtain a more accurate recognition result.

### 4.2. Gait Acoustic Signal AdaBoost Recognition Based on Enhanced GAF Methods and PCNN

While current vision-dominant gait recognition approaches have achieved remarkable success, their unignorable limitations become increasingly evident in complex environments [1,2,7,8,9,10,11,13]. These challenges often manifest as misinterpretations due to insufficient semantic understanding of specific frames, demonstrating that reliance on single-modality sensory data fundamentally restricts both comprehensive semantic extraction and global visual–audio physical characteristics under these circumstances [18,22,23,24]. Such constraints inevitably degrade abstract feature analysis in real-world applications. At the same time, some acoustic signal recognition has overemphasized time–frequency domain features while neglecting spatial characteristics and periodic variation patterns in multi-sensor frameworks. This oversight not only hampers the development of acoustic recognition methods but also significantly slows down the advancement of decision support mechanisms within our system.

To systematically address these negative aspects, an enhanced gait acoustic recognition method integrating a GAF-based method with a PCNN as base classifier components is developed. It is expected to focus more on spatiotemporal pattern learning, contextual feature awareness, and semantic relationship analysis.

Before employing the dual-input GAF-based graph representations, the Hilbert Transform is imperative. This arises from several factors. Firstly, our research focuses on continuous gait, and we are expected to capture the statistical regularities in movement patterns. Secondly, the envelope of acoustic signals contains key information, demonstrating that instantaneous amplitude fluctuations are critical. The Hilbert Transform serves as an essential tool to highlight changes in the energy spectrum across multiple gait periods. Moreover, it facilitates step-by-step capture of asymmetries, thereby laying the groundwork for subsequent analyses of statistical trends. There exists a close relationship between these elements and our dual-input GAF-based graph representations. For these reasons, we are quite convinced that they are inseparable within our system.

As for the gait acoustic signal recognition module presented in this study, a dual-input structure centered on the PCNN is established. In the dual-input base classifier, the upper branch (GADF-CNN) initially performs feature extraction through convolutional layers (by using a 5 × 5 convolution kernel, 32 filters, and an input/output channel ratio of 1:1; Stride = 2 and Padding = 2). This is followed by pointwise convolution (1 × 1 kernel, 32 filters) to enhance channel interactivity, and batch normalization is then applied along with the ReLU activation function, normalizing and activating across the 32 channels. Subsequently, max pooling (3 × 3, Stride = 2, Padding = 1) is adopted and leads to a fully connected layer that maps the flattened feature representation into a 64-dimensional vector space.

The lower branch (GASF-CNN) mirrors the same structure and parameters as the upper branch, with both branches independently processing their respective input data. In the end, a feature fusion process is conducted incorporating a multi-head attention mechanism. There is no doubt that the outputs from both branches are merged, and their features are concatenated. During this process, the multi-head attention mechanism (with 2 heads and key/query dimensions of 64) facilitates reconstruction. It is followed by a fully connected layer that maps the 64-dimensional features to an 8-dimensional logic output, utilizing the Softmax function to produce normalized class probabilities. Notably, the number of trainable parameters in the base classifier is approximately 3.7 million, with FLOPs around 0.3 billion. In the AdaBoost model, an early stopping strategy is employed, with the maximum number of iterations set to 15, resulting in an overall parameter count of approximately 33.5 million and FLOPs around 2.2 billion. Meanwhile, there is no doubt that the core recognition process can operate in an offline mode. These measures are adopted here to ensure that our system can perform gait recognition without requiring extensive training for each measurement.

**Figure 6:**
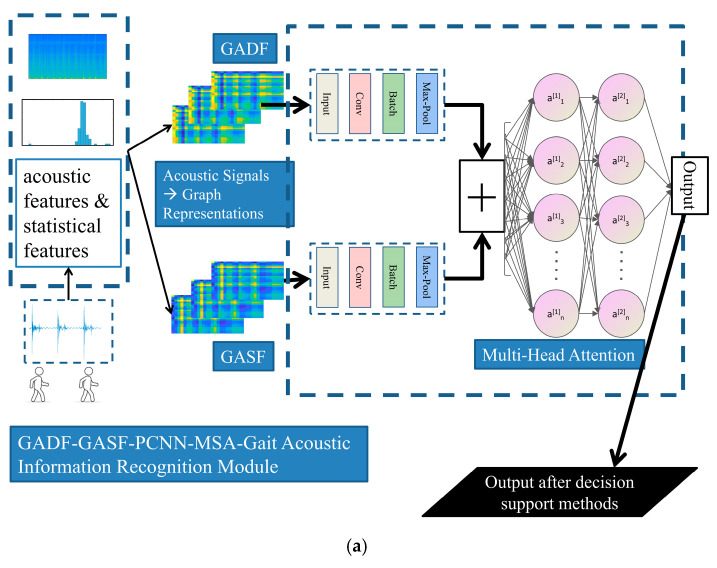

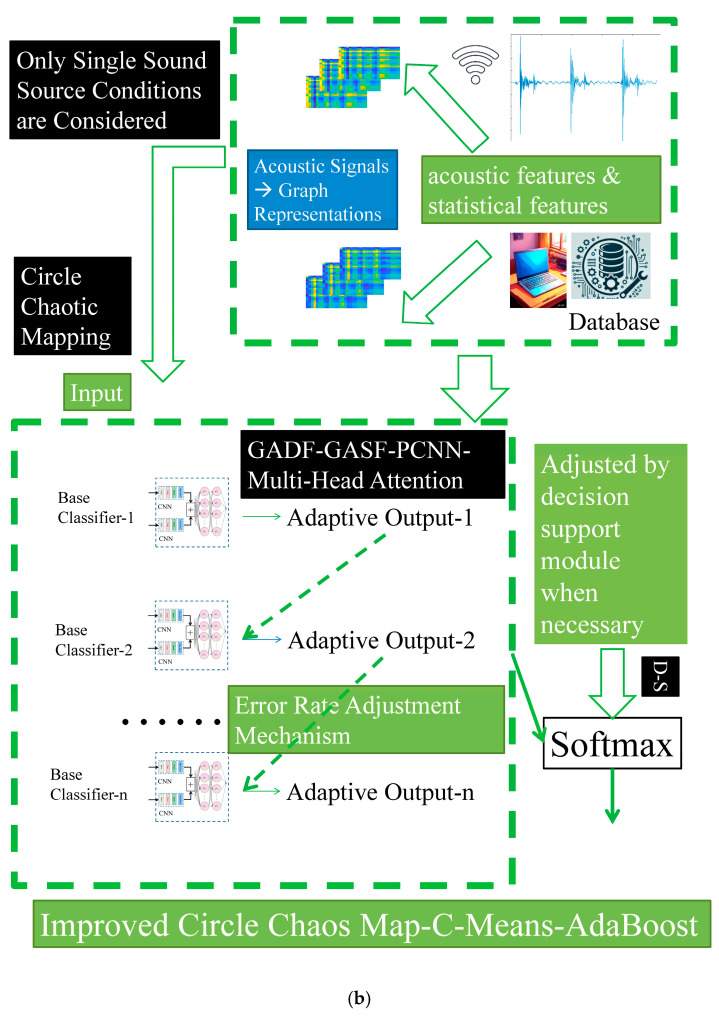
Audio information recognition module: (**a**) base classifier framework for audio information recognition module; (**b**) improved AdaBoost method for audio information recognition module.

As for the audio information recognition module, the base classifier framework and the entire system framework for gait acoustic signal recognition methods are shown in Figure 6a and Figure 6b, respectively.

The PCNN is adopted as the core component of the base classifier, bringing superior contextual learning capabilities compared to conventional CNN, RNN, or LSTM models. For periodic variation analysis and time–frequency characterization in time, each base classifier incorporates threshold-adaptive mechanisms. These enhancements strengthen the detection of dynamic anomalies in discrete periodic sequences while improving local connectivity awareness. The base classifier optimally integrates the enhanced GAF-based method. By leveraging both GADF and GASF representations across different modules, the system significantly improves recognition performance. These measures are adopted to effectively address several shortcomings in abnormal gait acoustic signals, including insufficient generalization in multi-scale coupling processes, weak periodicity–intensity correlation, and inadequate temporal coherence analysis. They not only enhance spatial feature perception but also adaptively balance global contextual understanding. At the output stage, a multi-head attention mechanism quantitatively evaluates feature contributions across different patterns. These acoustic signals are processed in parallel according to two different GAF-based transformations into 2D graph representations. They provide comprehensive and robust texture feature characterization within the base classifiers. Embedded within an improved AdaBoost framework, they are able to drive the process forward, combined with a c-means algorithm [4]. Ultimately, they lead to subsequent weight adjustments during further generalization periods.

By establishing this robust analytical foundation, our method simultaneously facilitates the subsequent D-SET-based decision support mechanism at both set-level and feature-level processing. It is bound to mitigate the heterogeneous semantic gap inherent in conventional approaches. All of these are intended to work together and obtain a more accurate result.

### 4.3. TTAO-D-SET Decision Support Mechanism for Gait Recognition Analysis

**Figure 7:**
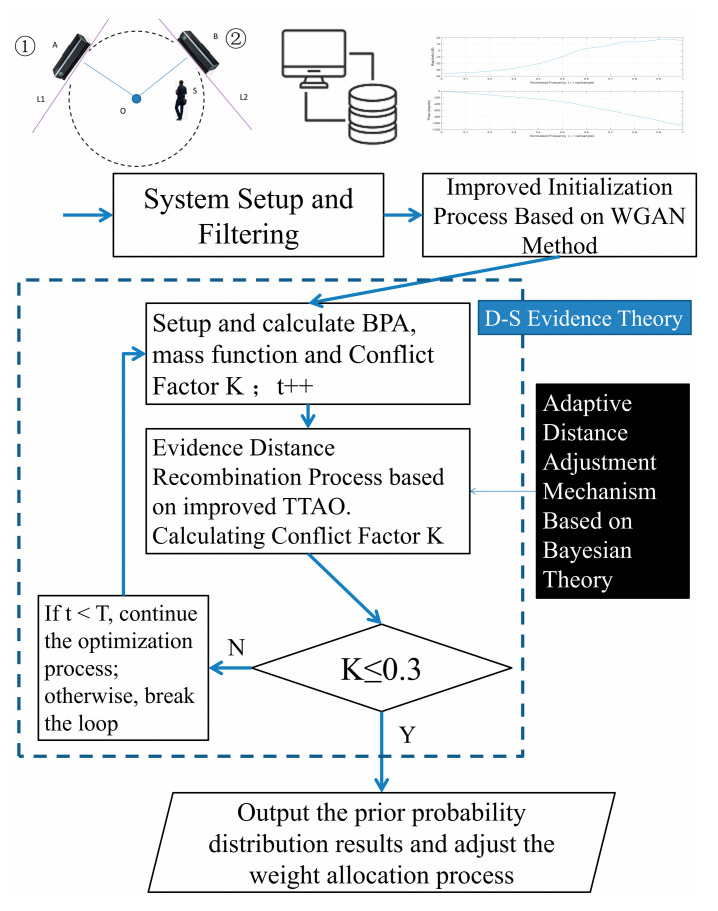
The decision support mechanism workflow.

Nowadays, multi-sensor systems encounter challenges in objectively assessing the applicability of various recognition modules within complex scenarios due to different factors [19,33,34,35]. They primarily arise from inadequate evidence distance comprehension and have limited capability of coping with occurrences during the training process. As for the incompatibility issues arising from an exclusive focus on dynamic weight updates in complex scenarios, the system incorporates a multi-sensor evidence distance representation. This adaptation proves particularly crucial for gait recognition tasks that require dynamic characteristic analysis and periodic variation detection. It iteratively updates the prior probabilities for each recognition module during this moment. These multi-source processing results enable quantitative analysis within the D-SET framework, ultimately achieving optimized evidence recombination with context-aware adjustments based on scenario-specific conditions. However, unpredictable concerns in D-SET may hinder the ability to mitigate negative impacts caused by extreme cases in BPA functions. This necessitates urgent improvements to evidence recombination processes in real-world situations. At the same time, we conduct a conflict factor calculation for each sub-task under evaluation. Among these, the conflict factor between the visual information recognition module and the gait acoustic signals recognition module in the “Falling” task is the lowest, with K = 0.09. In contrast, the conflict factor in the “Running Sounds” task is the highest, at 0.52. Based on these considerations, we set the static threshold for K at 0.3. Guided by these insights, our decision support mechanism is implemented, and its workflow can be described as follows (Figure 7).

To begin with, this mechanism optimizes evidence initialization by utilizing gait visual–audio information samples generated through an enhanced Wasserstein Generative Adversarial Network (WGAN) method. Different from the approaches from [2], this method employs Wasserstein distance as the loss function reference. Different from conventional GAN-based methods relying on cross-entropy theory or Jensen–Shannon (JS) divergence, our approach emphasizes distribution symmetry to more effectively evaluate the statistical characteristic differences among real gait patterns while mitigating gradient vanishing or explosion concerns. It can also strengthen the evidence distance generalization capability. The final integration with optimized visual and audio recognition modules facilitates robust recognition through adaptive weight adjustments. This adaptive weight adjustment process is then supposed to be further upgraded by the TTAO-based strategy.

To enhance the distinction of high-dimensional discrete sequences and improve the practical performance of evidence recombination based on generalized distance metrics, an improved TTAO updating strategy is introduced. It is specifically designed for scenarios with significant discrepancies in evidence support. The conventional TTAO operates based on two core mechanisms: generic aggregations and local aggregations. Generic aggregations generate new vertices by facilitating information exchange among different triangular topology units, while local aggregations seek new units according to geometric relationships. Together, they enhance the entire process of evidence recombination by balancing exploration and optimization. After integrating gradient descent optimization and N-dimensional distance generalization across evidence distributions, our strategy dynamically adjusts relevant decision weights for both visual and audio recognition modules. This leads to more context-specific decision making and improves its adaptability to varying scenarios. Furthermore, these improvements in evidence recombination facilitate more accurate gait recognition performance and are of great importance in maintaining discrimination capability in high-dimensional feature spaces. They are expected to guarantee robust evidence recombination under these circumstances. This process is denoted as TTAO(⋅) in subsequent formulations.

It can be distinctly noted that in our decision support mechanism, the TTAO refines the evidence recombination process through advanced distance generalization. Referring to Equations (3) and (4) in Section 2.2, the evidence optimization process for each recognition module can be regarded as a clustering procedure driven by prior probabilities. $\beta_{v}$ and $\beta_{a}$ are used to represent dynamically optimized weights for evidence bodies in the visual and audio recognition modules, respectively. The generalized referencing center is designated as G(i), while $P_{v}$ and $P_{a}$ represent the prior probabilities requiring optimization for visual and audio recognition modules. A Distance Sensitivity Indicator δ is used, and $\delta =10^{-6}$. The distance between visual and audio evidence bodies can be expressed as

(13) $$\left\{\begin{array}{c} TTAO(P_{v},P_{a})=\beta_{v}P_{v}+(1-\beta_{v})P_{a}\\ d(P_{v},G)=\sqrt{\sum_{i}{\left[P_{v}(i)-G(i)\right]}^{2}}\\ d(P_{a},G)=\sqrt{\sum_{i}{\left[P_{a}(i)-G(i)\right]}^{2}}\\ \beta_{v}=\frac{1}{1+e^{-\frac{d(P_{v},G)}{\delta }}}\\ \beta_{a}=\frac{1}{1+e^{-\frac{d(P_{a},G)}{\delta }}} \end{array}\right.$$

This strategy is subsequently integrated into the decision support module. It is expected to lead to quantitative analysis of module-specific performance limitations in adverse conditions and facilitate adaptive recognition method adjustments within our system. θ is adopted to represent the scenario-adaptive prior conditions, where the updated probability assignment results dynamically shape the module’s weight readjustment during the u-th iteration. This process can be described as

(14) $$P^{\left(u+1\right)}(\theta |E_{v},E_{a})=\frac{P^{\left(u\right)}(\theta )\cdot TTAO[P^{\left(u\right)}{\left(E_{v}|\theta \right)}^{w_{v}^{\left(u\right)}},P^{\left(u\right)}{\left(E_{a}|\theta \right)}^{w_{a}^{\left(u\right)}}]}{\sum_{\theta }P^{\left(u\right)}(\theta )\cdot TTAO[P^{\left(u\right)}{\left(E_{v}|\theta \right)}^{w_{v}^{\left(u\right)}},P^{\left(u\right)}{\left(E_{a}|\theta \right)}^{w_{a}^{\left(u\right)}}]}$$

Eventually, our method effectively mitigates the reliability shortcomings encountered in complex scenarios. Our updating strategy based on TTAO and its optimization techniques refine the evidence recombination process across multiple periods. By incorporating generalized distances between the geometric centroids of evidence sets into the decision support mechanism, our system implements adaptive weight adjustment for individual evidence bodies. It significantly improves recombination reliability through a flexible weighting mechanism and makes evidence closer to the geometric centroid receive higher weights, while those farther away are assigned progressively dynamic weights through an inertia weight nonlinear decrement [42]. This strategy demonstrably reduces negative impacts on the final results, thereby improving overall recognition accuracy. Furthermore, the generalized distance-based recombination quantitatively analyzes evidence trends to boost dynamic weight allocation. It is supposed to adaptively integrate both global evidence recombination and local BPA distribution optimization, effectively preventing excessive dominance by any single module while minimizing interference from sensor errors or noise. More gradual, stable weight adjustments are needed, and they can facilitate progressive decision support optimization. If K≥0.3 after this iterative process, the system is expected to terminate the decision making support in time. In the end, it successfully reduces decision support uncertainty and enhances gait recognition performance in complex situations.

## 5. Experiment and Analysis

Our core algorithm simulations are implemented by using MATLAB 2023b and Python 3.6.2, with TensorFlow 1.14.0 serving as the deep learning framework. Unity Hub 3.1.1 is also used (version: 2021.2.19f1). All executions are performed on a Lenovo Y9000P computer (Lenovo Inc., Beijing, China). It is equipped with an 11th Gen Intel^®^ Core™ i7-11800H processor and NVIDIA GeForce RTX 3060 GPU. During the experiment, the team seriously adheres to the rules of infectious disease prevention principles, daily management, safety regulations, and other relevant rules in our city. Meanwhile, there are no violations throughout the entire process. Subsequently, necessary measures and analysis are completed based on our self-made dataset and public datasets.

**Figure 8:**
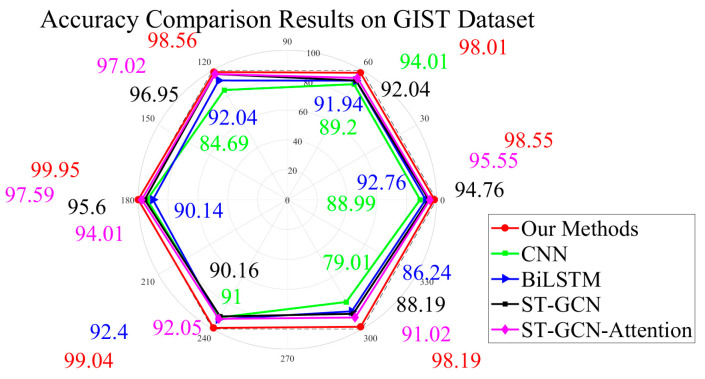
Accuracy comparisons of gait skeleton joint recognition methods.

The visual information recognition module is assigned a higher priority by default in our system. For our GASF-GEF-BiGRU-BiLSTM-MSA-AdaBoost recognition method, experiments are conducted referring to [2,4,5,6,7,43]. Evaluations are performed on the GIST dataset without the decision support mechanism. Six gait types are involved. They are normal gait, antalgic gait, lurch gait, steppage gait, stiff-legged gait, and Trendelenburg gait. Results are recorded in clockwise order starting from the 0° position below and shown as follows (Figure 8) [1,2,5,6,7,13].

All necessary evaluations in this manuscript are expressed as absolute percentage point differences. These comparative results clearly demonstrate significant performance improvements in the visual information recognition module. Obviously, our method achieves superior recognition accuracy for tasks involving normal gait, antalgic gait, lurch gait, steppage gait, and stiff-legged gait when compared with conventional CNN-based and BiLSTM-based methods. It can be seen that the GASF-GEF-BiGRU-BiLSTM-MSA-AdaBoost framework effectively enhances three core aspects of skeleton joint recognition. They are spatial information comprehension, semantic feature analysis, and contextual awareness. The dual-input structure successfully balances the multi-scale optimization process while leveraging its structural advantages. Their essential focus on asymmetric characteristics and anomalous events across gait periods proves to be effective, delivering accuracy improvements ranging from 1.53 to 19.18 percentage points over comparative methods in related recognition tasks. This also indicates that both the ST-GCN and ST-GCN-Attention methods effectively utilize the attention mechanism to allocate weights for long-range relationships in dynamic skeleton joint recognition tasks. On the one hand, they bring moderate improvements in static scenarios compared with other recognition methods [1,2,8,9,10,11,12,14]. However, due to projection transformations from the model, some latent factors that appear in the gait periods are overlooked. This may result in suboptimal performance in flexible orientation scenarios [4,13,41,42,43,44,45,46]. On the other hand, some methods fail to highlight envelope information and spatial structures during the main process, leading to ambiguity in distinguishing between the Swing phase and the Stance phase. Different from them, our proposed method effectively addresses these shortcomings, which is particularly evident in the Trendelenburg gait recognition sub-task. In detail, compared with the ST-GCN and ST-GCN-Attention methods, our proposed method improves accuracy by 10.01 percentage points and 7.17 percentage points, respectively. They display a significant enhancement.

**Figure 9:**
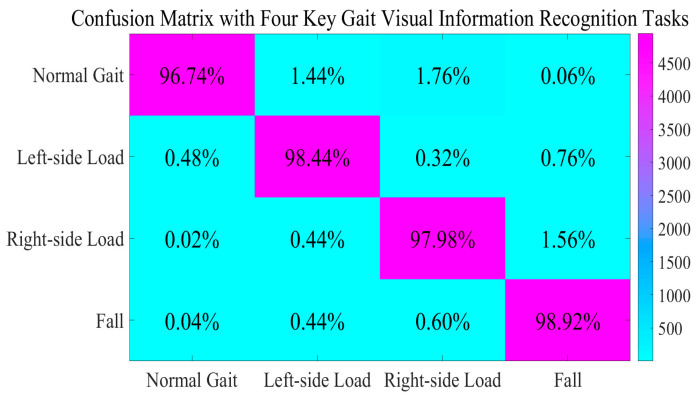
Comparisons of four key visual information recognition tasks.

We constructed our own dataset for dual-Kinect V2 systems. It consists of skeleton joint data from gait, falls, and daily activities according to [2,3,4,7,15,43,44]. Some necessary principles are also considered [37,38]. In our experiments, we invited six volunteers (five males and one female) to obtain gait data under loaded conditions. All participants had no difficulties in walking or visual impairments in their daily lives. We instructed them to perform data collection while walking on one leg with a 6 kg sandbag. These measures are intended to better simulate the walking conditions experienced by certain patients. In specific scenarios, both the height of the Kinect V2 from the ground and its pitch angle could be adjusted. During data collection, participants were encouraged to complete several entire gait periods (usually 2–8 periods). After comprehensive data aggregation and necessary preprocessing, we selected 20,000 subsamples per task with a 3:1 training-to-testing split ratio. These recognition tasks are defined as **task 1 (normal gait), task 2 (left-side load), task 3 (right-side load), and task 4 (fall)**. For all of the confusion matrices presented in this manuscript, they are used to calculate the proportion of correctly identified tasks within each sub-task through row-wise analyses. This approach provides a more effective reflection of the recall rate and its statistical characteristics. The following table reveals the recognition results of the GASF-GEF-BiGRU-BiLSTM-MSA-AdaBoost method, displaying the proportion of correctly recognized instances relative to the total number of instances for each task (Figure 9).

As illustrated in Figure 9, our proposed method not only demonstrates excellent performance in the normal gait recognition task but achieves remarkable results in recognizing irregular gait patterns randomly, as well. It can be distinctly noted that in scenarios characterized by flexible visual information, the methods within this module can effectively balance the essential requirements of static action recognition tasks that emphasize both time–frequency domain information and spatial information. At the same time, they also finish the analysis of periodic trends and abstract feature understanding in dynamic action recognition tasks. As a result, the overall performance is outstanding. A subsequent comparison of our method with other methods is presented in the following table (Table 1).

According to the results presented in Figure 9 and Table 1, several conclusions can be drawn. Firstly, some attention-based approaches and multi-scale methods exhibit excellent performance. They effectively cope with the inherent limitations of RGB stream methods in managing the incompatible learning capabilities for contour and texture features across different granularities. However, their spatial information processing significantly constrains the adaptive perception of skeleton topology structures. In contrast, the AdaBoost-based framework achieves comparable recognition performance through balanced multi-scale feature integration via optimized base classifier weight adjustments. Notably, our method outperforms the approach in [9] by 2.06 percentage points in flexible orientation scenarios and surpasses the multi-scale feature methods in [1,13] by 2.42 and 3.87 percentage points, respectively. Secondly, the results of the ablation experiments concerning our GAF-based representations and Circle Chaotic Mapping demonstrate that they play a crucial role in the multi-scale representations within our system. Without the GAF-based approaches, our core method exhibits significant weaknesses in simulating dynamic sequences and analyzing complex temporal patterns. It fails to adequately summarize multi-scale gait dynamic information, which hinders our proposed method from fully leveraging the complex feature relationships and sequential information inherent in skeleton joint data. This limitation is significantly influenced by the implicit dependencies and feature interactions present during each gait period, ultimately amplifying the disadvantages of the AdaBoost-based model and leading to its performance being inferior to several Transformer-based methods. This underscores the inseparable nature of the various modules within our system. However, some methods that overemphasize semantic clue perception fail to deliver the expected performance in single-person scenarios. This suggests that their semantic mining functionality may potentially encroach on the capacity for detailed spatial information comprehension, which sometimes proves harmful to gait skeleton joint recognition. As for the Graphormer method mentioned above, it offers substantial advantages in skeleton joint gait recognition. Its foundation is the Graph Neural Network (GNN) [46]. Its strong analytical capabilities concerning skeleton joint structures help it effectively capture the correlations among gait skeleton joints. However, ineffective hyperparameter combinations may significantly limit the practical effectiveness of Graphormer. Furthermore, its high computational complexity often restricts its adaptability. As for the TA-CNN methods, some conclusions can be given. On the one hand, the CNN module in TA-CNN plays an essential role in feature comprehension for skeleton joint sequences. It effectively captures crucial information regarding the positions and angles of core joints during the gait period. Simultaneously, its Transformer module further enhances the perception of spatiotemporal information, reinforcing the relevance of different gait periods. On the other hand, it should be noted that the contexts of eye movement detection in multi-person scenarios, as applied in TA-CNN, do not entirely align with the context of this study. Clearly, the GAF representation approach utilized in this study works in close conjunction with the recognition model, highlighting the asymmetry between different gait phases and thereby improving the differentiation of abnormal gaits. In contrast, the TA-CNN model has struggled to effectively address the representational differences required for skeleton joint feature representations, and its inability to appropriately accommodate the structured characteristics has limited its practical effectiveness. We also present a comparison of several Transformer-based approaches, and more conclusions are coming out. It is undeniable that Transformer-based methods significantly enhance the ability to synthesize dynamic time–frequency domain clues and help to comprehend multi-scale features. This indicates that they provide an objective framework better suited for uncovering subtle clues. When in the gait skeleton recognition tasks, the capability to capture long-range relationships among periodic skeleton joint data is sufficiently robust, allowing for analyzing implicit associations among multiple core skeleton joints across different periods. Furthermore, some attention mechanism characteristics are beneficial in determining feature contributions, facilitating adaptive weight adjustments in subsequent stages. This adaptability makes the gait representations more applicable to multiple subjects. However, the Transformer-based approach also has its limitations. In our system, the framework and feature extractions are specifically designed to emphasize the asymmetry of skeleton joints, which means that high computational complexity is not tolerable for our model. In contrast, this is not the case for Transformer-based methods, where such complexity is almost inherent. Such limitations hinder their effectiveness in contexts where feature dimensions are low. As a matter of fact, our concave pentagon methods mentioned above are adopted to highlight the dynamic relationship between the upper and lower body in the statistical approximation. This method reduces the model’s necessity to uncover the relationships and detailed analysis of the temporal connectivity characteristics among skeleton joints. In conclusion, there is no doubt that the AdaBoost-based method, in close conjunction with a feature engineering approach, is better aligned with the essential requirements of this situation. Comparative results demonstrate that our method outperforms others in advantageous situations. But, it should be noted that the experimental environment is artificially constructed to minimize conditions under which visual information might induce additional cognitive ambiguity. While this setup allows certain methods to achieve relatively impressive recognition results, it raises concerns regarding their generalizability to more challenging scenarios.

**Figure 10:**
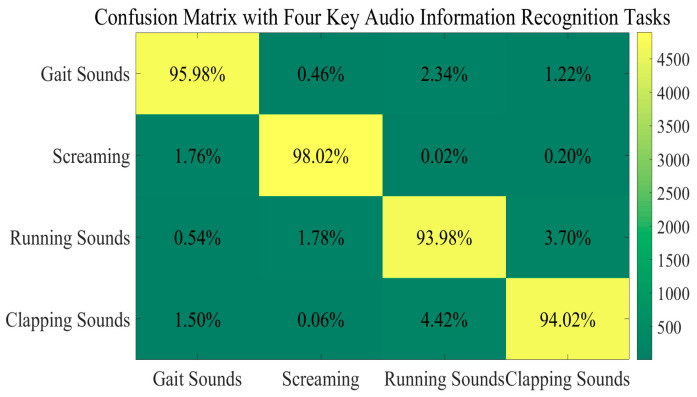
Comparisons of four key audio information recognition tasks.

As for gait recognition, some methods may fail to fully leverage their strengths in advanced spatial feature perception. Instead, they are becoming more susceptible to dynamic variations caused by different temporal–frequency resolutions. While gait-related visual and audio information emphasizes periodic patterns and the asymmetry of abnormal gaits, their focal points differ significantly. The former primarily focuses on spatial structural changes, whereas the latter emphasizes analytical processes incorporating time–frequency domain characteristics. This means that some approaches well-suited for gait visual recognition may not always be suitable for gait acoustic signal recognition modules. Meanwhile, some acoustic scene classification models struggle to perform well due to limitations in unimodal information generalization. In multi-sensor systems, this may manifest as significant performance disparities across different modules. For instance, walking recognition tasks tend to rely more on audio information than visual information compared with running recognition tasks. The situation differs for walking vs. clapping recognition tasks. Accordingly, we conducted comparative experiments on **gait sounds, screaming, running sounds, and clapping sounds** with reference to [23,24,47,48,49] and, partially, our own datasets. Each category contains 20,000 samples. By calculating the ratio of correctly identified instances to the total number per task, the following comparisons are obtained across the four key audio information recognition tasks (Figure 10).

Some other conclusions can clearly be drawn. First and foremost, our GADF-GASF-PCNN-MSA-AdaBoost method in our audio recognition module demonstrates competitive recognition performance. It effectively mitigates the disadvantage associated with single-module approaches in complex real-world situations. By adopting GAF-based methods and a PCNN architecture, our method achieves a balanced reconciliation between time–frequency domain interpretation and abstract feature extraction. Furthermore, it facilitates a granular analysis of envelope information by exploiting periodic gait characteristics buried in acoustic signals, thereby ensuring robust and adaptable recognition. These synergistic advantages collectively enhance contextual acoustic signal perception, as evidenced by a 98.02% recall rate for the screaming task, where other non-periodicity-aware methods significantly underperform. It is clear to see that our method maintains consistent efficacy even in challenging scenarios. For human-made pathological running sounds and clapping sounds, it achieves 93.98% and 94.02% recall rates, respectively. Additional comparative studies against other recognition methods further validate its superiority. They are presented below (Table 2).

Some other conclusions can also be drawn from Figure 10 and Table 2. Firstly, our method successfully captures the time–frequency characteristics of single-source sounds within specific periods while maintaining segmentation capabilities. Although some methods demonstrate certain multi-scale learning effectiveness for time–frequency information, they exhibit latent limitations in processing non-stationary characteristics. In contrast, our dual-input structure achieves a comprehensive representation of both multi-scale gait features and transient properties by adding statistical feature extractions. Secondly, they also reveal that approaches effective in other domains (e.g., mechanical anomaly detection [22,32]) may underperform in this situation. Those gait-related acoustic signals exhibit more controllable patterns compared to mechanical anomalies where periodicity is not universally required. Furthermore, while both domains are influenced by material properties, those acoustic signal analyses often rely more on rigid assumptions that are inadequate for handling the dynamic variations that appear in gait periods. Our GAF-based method significantly enhances subtle feature extraction across temporal scales with its dual-input structure. Furthermore, 98.05% and 98.93% accuracies are achieved for gait sound and screaming tasks, respectively, with corresponding recall rates of 95.98% and 98.02%. These results also indicate superior time–frequency pattern generalizability in these scenarios. In comparison, running sound and clapping sound recognition achieves slightly lower accuracies (96.80% and 97.22%) and recall rates (93.98% and 94.02%). This reveals persisting challenges in accounting for adopting GAF-based approaches alone. As for the SVQ-MAE method, it achieves the best recognition outcomes in gait-related acoustic signal recognition tasks. This model utilizes a Transformer-based encoder to construct contextual representations of voice signals [49]. This effectively extracts temporal features and helps to fully capture key information, such as periodic characteristics and frequencies. On this basis, it improves the recognition accuracy. However, it is important to note that the core focus is quite different between gait acoustic signal recognition tasks, which emphasize multi-period feature capture, and existing pattern recognition tasks. Consequently, specific adaptations and adjustments are urgently required in this area. Meanwhile, compared to the core methods of our gait acoustic signal recognition module, the high computational complexity of this approach presents lots of conflicts with the decision support mechanisms in our system. They need to be considered carefully.

At the same time, these illustrations also objectively highlight the limitations of methods based on a single recognition module in executing recognition tasks within complex scenarios. There is no doubt that these methods are unable to fully leverage the advantages of cross-modal feature perception. During gait recognition periods, this indirectly suggests that visual information holds an advantageous position in spatial feature perceptions, while related audio information recognition approaches excel in time–frequency domain collaborations. Following this, it powerfully exposes that a multi-sensory system can greatly enhance performance through dynamic information fusion or scene discrimination mechanisms. Therefore, it is essential to compare the actual recognition effects before and after the introduction of the decision support mechanism in challenging scenarios.

**Figure 11:**
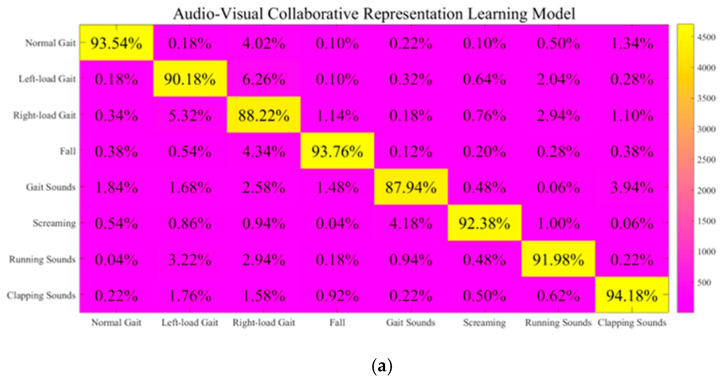

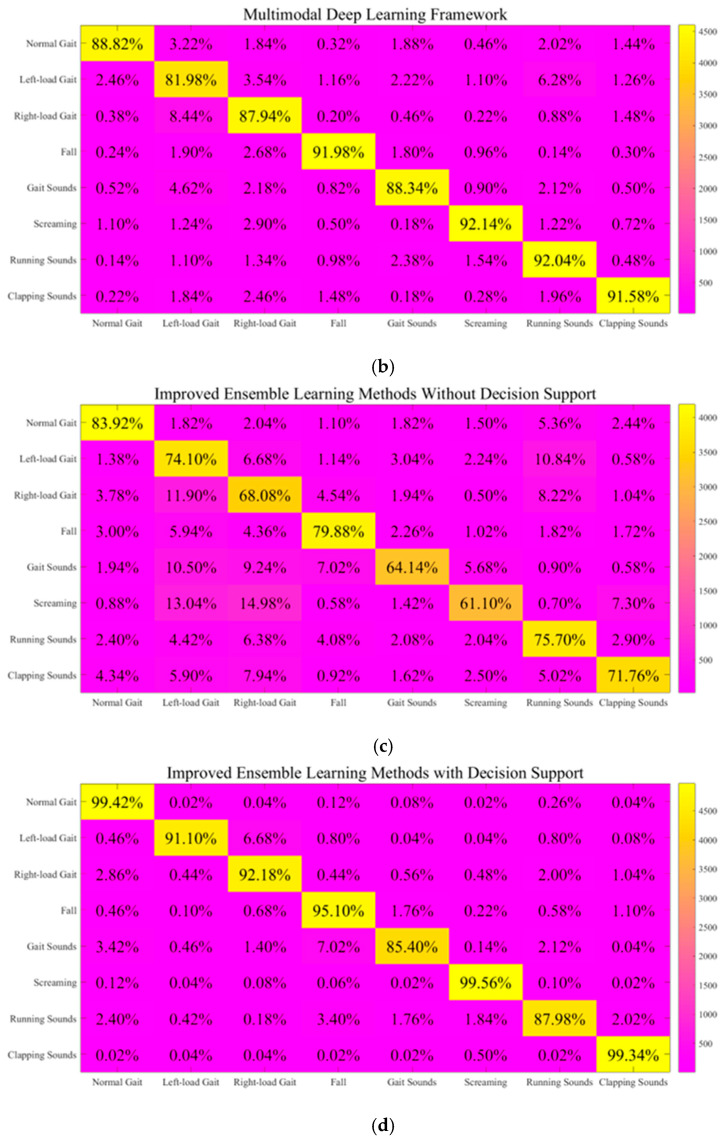
Comparison results of eight challenging recognition tasks: (**a**) comparison results of audio–visual collaborative representation learning model; (**b**) comparison results of multimodal deep learning framework; (**c**) comparison results of improved ensemble learning methods without decision support; (**d**) comparison results of improved ensemble learning methods with decision support.

Several projects have developed multimodal fusion frameworks for multi-sensor information processing, with the approaches from [18,44] serving as representative instances. Different from them, our TTAO-D-SET decision support mechanism is able to effectively adjust the recognition module weights in challenging situations, thereby achieving adaptive module optimization. Accordingly, we conducted a detailed analysis in eight challenging scenarios. The eight tasks were set as **task 1 (normal gait), task 2 (left-load gait), task 3 (right-load gait), task 4 (falling), task 5 (gait sounds), task 6 (screaming), task 7 (running sounds), and task 8 (clapping sounds)**. To better illustrate the advantages of the decision support mechanism, we ensured that all eight challenging tasks occurred randomly, preventing each tested framework from functioning exclusively within its specialized scenario. Subsequently, we compared four frameworks: the audio–visual collaborative representation learning model [18], the multimodal deep learning framework [44], the enhanced ensemble learning framework without decision support, and the enhanced ensemble learning framework with a decision support mechanism. We conducted a detailed comparison of our system’s performance before and after the introduction of the decision support mechanism. Below, we present the average results from all experiments (10 test runs), which were obtained after conducting 10 test runs. We believe that the recall rates reflect the influence on missed detection instances in gait recognition. This is obviously more important than false detection in this context. The results can be shown as follows (Figure 11 and Table 3).

According to the discussions mentioned above, the following conclusions can be clearly given. First of all, all methods exhibit high levels in both the normal gait and falling tasks. Unlike some falling recognition studies, our methods place greater emphasis on spatial structure comprehension. This helps a lot across many tasks. On the one hand, a comprehensive comparison of these results displayed in Figure 11a–d reveals that the GAF-based method without the TTAO-D-SET decision support mechanism performed relatively poorly in these tasks. It can be found that methods from [18,44] and our TTAO-D-SET-optimized approach demonstrate improvements of 19.18, 17.01, and 21.42 percentage points in average recall rates, respectively, across all eight tasks. The average recognition accuracy is improved by 4.80, 4.26, and 5.36 percentage points, while the average precision is increased by 17.95, 15.73, and 20.05 percentage points, respectively. Further analysis clearly showcases that the most significant improvements occur primarily in scenarios where audio information holds an advantageous position. After incorporating the decision support mechanism, our method achieves average recall rate improvements of 2.24 and 4.41 percentage points compared with [18,44] in complex scenarios, with corresponding overall accuracy improvements of 0.56 and 1.10 percentage points. This shows that our method can effectively handle flexible recognition tasks with relatively lower miss rates when combined with the decision support mechanism. On the other hand, the models proposed in [18,44], as well as our approach with decision support, each exhibit their remarkable strengths in these tasks. By leveraging their multi-scale information perception processes, the methods from [18,44] can balance visual and audio feature perception in specific scenarios. This capability helps the models reason about differences in prior conditions within test scenarios to some extent and seek adaptive matching of multi-scale feature spaces that emphasize prior relationships. Consequently, this promotes the discovery of underlying multimodal correlations and semantic consistency. This is particularly evident in specific tasks. For instance, in the normal gait, falling, gait sound, and running sound tasks, the approach from [18] achieves higher recognition accuracy by 0.04, 0.82, 0.07, and 0.31 percentage points compared to our method. In addition, the method from [44] outperforms our approach by 0.41 percentage points in the falling task. These findings confirm that the core methods employed in [18,44] retain their advantages in specific situations.

Furthermore, our recognition framework powerfully unveils unique advantages with a decision support mechanism. On the one hand, the improved data-driven reasoning process enabled by this mechanism allows our method to excel, particularly in scenarios exhibiting periodic regularities. This is evident in our superior ability to accommodate task-specific requirements through necessary upgrades and adaptive adjustments when compared with the approaches outlined in [18,44]. Our framework facilitates more effective visual information processing through flexible spatial analysis while maintaining optimal acoustic signal processing that emphasizes time–frequency characteristics and relevant spatial correlations. What is more, our system also successfully integrates the preprocessing methods for skeleton joint and acoustic signals mentioned above. They are supposed to collectively enhance overall recognition performance and decision support capability. On the other hand, experimental results reveal prominent limitations in [18,44] stemming from their strong dependence on training data characteristics. Their deep representation learning processes tend to overfit existing patterns in the dataset, which consequently weakens their generalization capability within individual modalities. This inherent factor creates implicit dependencies on prior conditions that ultimately limits recognition performance in related tasks. Higher-dimensional feature representations are not always suitable for recognition methods, and these approaches may incorrectly allocate excessive training weight adjustments. After all, this mismatch leads to inefficient model training processes and suboptimal recognition performance, which is particularly evident in four challenging tasks: left-load gait, right-load gait, screaming, and clapping sounds. Other methods may struggle with these tasks due to limited time–frequency analysis capabilities under periodic generalization requirements and multi-granularity information modeling. Next, quantitative comparisons demonstrate our method’s effectiveness over [18], with recall rate improvements of 0.92, 3.96, 7.18, and 5.16 percentage points, accuracy improvements of 1.62, 2.20, 0.89, and 1.02 percentage points, and F1-score increases of 6.07, 7.92, 3.70, and 4.07 percentage points across the four tasks, respectively. When compared with [44], the advantages are even more pronounced, as recall rates improve by 9.12, 4.24, 7.42, and 7.76 percentage points, accuracies improve by 3.75, 1.51, 1.21, and 1.20 percentage points, and F1-scores improve by 14.35, 5.74, 4.93, and 4.93 percentage points. These results confirm that our system provides substantially enhanced gait recognition capability through our AdaBoost-based methods and an effective decision support mechanism. It turns out to be a critical advancement with significant practical implications in real-world applications.

As a final point, our method achieves adaptive visual and audio information recognition module adjustments based on improved TTAO-D-SET decision support. It enhances gait visual–audio information recognition performance across different situations, and it has been empirically validated as effective when integrated with other modules within our system. Quantitative evaluations reveal consistent improvements over the methods from [18,44], with absolute increases of 0.56 and 1.10 percentage points in average recognition accuracy, 2.10 and 4.32 percentage points in precision, 2.24 and 4.41 percentage points in recall rate, and 2.08 and 4.26 percentage points in F1-score, respectively. These results demonstrate that the connection between our recognition modules and the decision support mechanism effectively mitigates the negative impacts on gait recognition. Alongside this, it should be noted that the methods [18,44] still exhibit excellent performance within their respective domains. This finding leads to promising directions for future research, particularly in deep-level multimodal information interactions within multi-sensor systems. Last but not least, it is crucial to dedicate our efforts to reducing the computational complexity of the methods presented in this manuscript. This will be useful for cross-platform deployment.

## 6. Conclusions and Future Work

Human gait recognition has become a paramount component in various Internet of Things systems. In these days, many researchers have predominantly focused on visual gait information and obtained notable results. Some shortcomings are gradually exposed, and this means necessary upgrades are needed. To tackle these, we establish a multi-sensor system based on AdaBoost-based ensemble learning and develop our core methods for visual and audio information recognition. Furthermore, we also introduce a decision support mechanism based on an improved TTAO-D-SET framework. It leads to dynamic module weight adjustments and enhances recognition performance. With this as a springboard, some conclusions can be drawn from our experiments.

As for the visual information recognition module in our system, our GASF-GEF-BiGRU-BiLSTM-MSA-AdaBoost method focuses more on the representations of periodic patterns and spatial characteristics, thereby enriching multi-scale feature comprehensions. Experimental validation of the GIST dataset demonstrates its superior performance. It achieves 98.72% recognition accuracy and surpasses all current state-of-the-art approaches. Furthermore, more evaluations using our own dataset yield even more impressive results, attaining an accuracy of 99.01% for four key gait recognition tasks. These results are sufficient to demonstrate the powerful performance of our methods in this module.

As for the audio information recognition module in our system, we integrate time–frequency information comprehension with statistical characteristic processing. On this basis, our GADF-GASF-PCNN-MSA-AdaBoost method for gait acoustic signal recognition is evaluated by using our own dataset, demonstrating exceptional performance, with a recognition accuracy of 97.75% across four key audio information recognition tasks. These results not only confirm the substantial ability to simultaneously manage multi-scale information perception and time–frequency analysis; they also provide a solid foundation for subsequent decision support mechanisms, as well.

As for the decision support mechanism, an improved TTAO-D-SET method is adopted. This mechanism is considered effective for the weight adaptive adjustments across various modules within our system. The close collaboration among the visual information recognition modules, audio information recognition modules, and decision support mechanisms significantly enhances the entire recognition performance. The experiment results confirm that our entire framework outperforms all others in eight challenging recognition tasks.

The shortcomings exposed in this manuscript will serve as the standpoint of our research in the future. Firstly, our system is designed and constructed on Kinect V2 devices. While these devices offer considerable versatility, they have recently revealed some limitations in specific scenarios. Therefore, we are about to explore more suitable equipment to build up our new system. Meanwhile, exploring the most rational layout using tools like the Unity engine constitutes a significant component. Adopting the most reasonable deployment methods for multi-sensor devices and investigating their general principles and broader implications will inevitably become a key focus in response to the requirements of gait recognition contexts. By exploring more effective developmental arrangements, we aim to further leverage the advantages of the equipment while minimizing the impact of negative factors that are challenging to quantify. More analysis regarding gait visual information and gait-related audio information in noisy environments also needs to be addressed in future research. As for the gait acoustic signals, multi-scale feature extraction methods are needed. So, the applicability of spectrogram Transformers in gait audio signals will be a potential topic for our system. They are supposed to enable our system to further extract the latent emotional clues embedded in audio signals based on user historical data. Accompanied by advancements in hardware capabilities, they are expected to play a more significant role. Secondly, our core method focuses only on single-person scenarios, leaving multi-source audio–visual recognition untouched. So, we are also going to incorporate clock synchronization methods and priority allocation logic. They are essential when our system is used in more complex situations. Thirdly, the development of information transmission protocols between multi-sensor devices for scheduling priority allocation will also become an important focus for upcoming investigations. Fourth, it is evident that there is an urgent need to deepen the research and development of gait recognition in multi-person scenarios for forthcoming studies. To effectively develop gait recognition methods in multi-person scenarios, it may be necessary to integrate confidence levels or segmentation techniques to prioritize task-allocation mechanisms. In addition, some Large Language Models may provide substantial support and further expand the adaptability of our system. At the moment, it is evident that the visualization and interpretation of low-dimensional features of gait using Transformer-based or other approaches, along with methods for weight readjustment specifically tailored to this context, will emerge as a significant topic next season. Last, but equally important, our current approach still struggles to completely eliminate the tendency to disproportionately amplify the weights of certain sensors during the training process. For that reason, Fuzzy Inference Theory or Game Theory may be indispensable for our decision support mechanism. They are expected to broaden its applicability in the next stage.

## Figures and Tables

**Table 1:** Comparison results of gait visual information recognition methods.

Methods	Accuracy
SVM	64.29%
SVM-AdaBoost	79.48%
CNN	76.29%
Multi-Head CNN [14]	88.14%
ST-GCN	88.95%
ST-GCN-Attention	90.13%
GAN-BERT [2]	92.98%
Attention-Guided and Topology-Enhanced Shift GCN [11]	95.15%
Leveraging multimodal deep learning [44]	94.55%
Twin-Tower [7]	92.55%
Zoom Transformer [13]	95.14%
Graphormer [46]	95.58%
Our method without GAF-based representations	88.92%
Our GASF-GEF-BiGRU-BiLSTM-MSA-AdaBoost Without Circle Chaotic Mapping	92.63%
TA-CNN [45]	96.04%
Select–Assemble–Normalize Graph Convolutional Networks [37]	96.24%
Multi-stream+multi-scale dilated spatial–temporal graph convolution network [1]	96.59%
Global spatio-temporal synergistic topology [9]	96.95%
Multi-scale sampling attention Graph Convolutional Networks [38]	98.84%
Our GADF-GASF-PCNN-MSA-AdaBoost (used for gait acoustic signal recognition methods)	93.18%
Our GASF-GEF-BiGRU-BiLSTM-MSA-AdaBoost	99.01%

**Table 2:** Comparison results of gait-related acoustic information recognition methods.

Methods	Accuracy
SVM	69.14%
CNN	73.48%
SVM-AdaBoost	79.12%
LSTM	70.79%
BiLSTM	84.94%
Multi-Scale Audio Spectrogram Transformer [47]	96.19%
Temporal Transformer-Based Autoencoder [22]	77.29%
BEAT-BiGRU-frequency-dynamic convolutional network [23]	92.98%
Perturbed Residual Recurrent Neural Network [24]	96.98%
SVQ-MAE [49]	98.99%
Our GASF-GEF-BiGRU-BiLSTM-MSA-AdaBoost (used for gait skeleton recognition methods)	94.99%
Our GADF-GASF-PCNN-MSA-AdaBoost	97.75%

**Table 3:** Comparison results of eight challenging recognition tasks.

Methods	Accuracy (%)	Precision (%)	Recall (%)	F1-Score (%)
[18]	97.88%	91.78%	91.52%	91.60%
[44]	97.34%	89.56%	89.35%	89.42%
Our methods (**without** decision support)	93.08%	73.83%	72.34%	72.56%
Our methods (**with** decision support)	98.44%	93.88%	93.76%	93.68%

## Data Availability

We are unreservedly willing to provide research data or key codes mentioned in this manuscript. If necessary, please contact Rui-Xiang Kan via email (bbklasnic@glut.edu.cn) to obtain the Baidu Netdisk (Baidu Cloud) URL link and then download the files you need. The files are provided in a zip file, and some illustrations are also added. If the link becomes inaccessible, please e-mail us in English or in Chinese to obtain the new link. We will answer you as soon as possible.
